# Different Types of Vaccines against Pestiviral Infections: “Barriers” for “*Pestis*”

**DOI:** 10.3390/v15010002

**Published:** 2022-12-20

**Authors:** Mengqi Yuan, Xiaoke Yang, Xin Zhang, Xiaotian Zhao, Muhammad Abid, Hua-Ji Qiu, Yongfeng Li

**Affiliations:** 1 State Key Laboratory of Veterinary Biotechnology, Harbin Veterinary Research Institute, Chinese Academy of Agricultural Sciences, Harbin 150069, China; 2 College of Animal Science and Veterinary Medicine, Henan Institute of Science and Technology, Xinxiang 453003, China; 3 Tianjin Key Laboratory of Agricultural Animal Breeding and Healthy Husbandry, College of Animal Science and Veterinary Medicine, Tianjin Agricultural University, Tianjin 300384, China; 4 Viral Oncogenesis Group, The Pirbright Institute, Ash Road, Pirbright, Woking, Surrey GU24 0NF, UK

**Keywords:** pestiviruses, live attenuated vaccines, marker vaccines, multivalent vaccines

## Abstract

The genus *Pestivirus* of the family *Flaviviridae* mainly comprises classical swine fever virus (CSFV), bovine viral diarrhea virus 1 (BVDV-1), BVDV-2, border disease virus (BDV), and multiple new pestivirus species such as atypical porcine pestivirus (APPV), giraffe pestivirus, and antelope pestivirus. Pestiviruses cause infectious diseases, resulting in tremendous economic losses to animal husbandry. Different types of pestivirus vaccines have been developed to control and prevent these important animal diseases. In recent years, pestiviruses have shown great potential as viral vectors for developing multivalent vaccines. This review analyzes the advantages and disadvantages of various pestivirus vaccines, including live attenuated pestivirus strains, genetically engineered marker pestiviruses, and pestivirus-based multivalent vaccines. This review provides new insights into the development of novel vaccines against emerging pestiviruses, such as APPV and ovine pestivirus.

## 1. Introduction

The genus *Pestivirus* (from the Latin *pestis*—plague), belonging to the family *Flaviviridae*, is responsible for infectious diseases in swine, cattle, sheep, goats, and other domestic and wild animals. According to the recent reclassification of the genus *Pestivirus* by the International Committee on Taxonomy of Viruses (ICTV), the pestivirus taxonomy resulted in the demarcation of eleven species designated pestiviruses A through K. Due to the increasing number of diverse pestiviruses, the taxonomy of the genus *Pestivirus* was revised in 2017 based on nucleotide or amino acid sequence distances of complete coding sequences, combined with antigenic differences, natural host ranges, and pathology. The number of pestivirus species was expanded to nineteen by identifying eight new species designated pestiviruses L through S, including atypical porcine pestivirus (APPV), giraffe pestivirus, antelope pestivirus, HoBi-like pestivirus, Bungowannah virus, and Linda virus [1,2,3,4,5,6,7,8,9]. Pestiviruses contain a single-stranded, positive-sense RNA [10,11,12]. The sizes of pestiviral genomes are approximately 12.3 kb, harboring a 5′ untranslated region (UTR), a single long open reading frame (ORF), and a 3′ UTR. The ORF encodes four structural and eight nonstructural proteins that are processed by viral and cellular proteases [13,14,15,16,17].

The pestivirus-associated epidemics have caused significant economic losses in many countries with intensive animal husbandry. The prevention and treatment of pestiviral infections have become quite challenging due to the lack of strict host specificity and the considerable variability among pestiviruses [15,18,19,20]. Live attenuated vaccines (LAVs) have been developed to prevent and control pestiviral infections. Some of the vaccines are commercially available. However, most LAVs do not allow differentiation by serology of infected from vaccinated animals (DIVA), complicating the eradication of the related disease [21]. Marker vaccines are highly preferred since they can avoid the economic loss caused by the ethically and economically disadvantageous slaughter and culling during the implementation of the “stamping-out policy” for CSF [22]. For the eradication of CSF, different types of marker vaccines that comply with the DIVA principle have been developed based on the E2 protein or chimeric pestiviruses. Additionally, co-infections of pestiviruses with other viruses, such as pseudorabies virus (PRV), African swine fever virus (ASFV), porcine parvovirus (PPV), and porcine circovirus type 2 (PCV2), pose a significant threat to the swine industry [23,24,25,26,27,28]. Therefore, the development of a multivalent vaccine is necessary for the prevention of co-infections.

In this review, we summarize the development of various pestivirus vaccines and discuss in detail the advantages and disadvantages of these vaccines in terms of the safety, efficacy, and feasibility to express foreign genes in the backbone of pestiviruses.

## 2. Advances in Vaccine Approaches against Pestiviral Infections

Vaccination is an attractive strategy for controlling pestiviral infections. Various vaccines against the pestiviral infections have been widely used in the field for several decades [29,30,31]. However, there are still some important issues in the production and application of pestivirus vaccines, including safety, productibility, and the duration of immunity [32,33,34]. Current pestivirus vaccines have inherent advantages and disadvantages (Table 1). Novel pestiviral vaccines need to be developed due to the shortcomings of existing products.

### 2.1. LAVs against CSF

There are a variety of LAVs developed against CSF through continuous passages in non-susceptible cells or hosts, including the Chinese strain (C-strain), Riems strain, GPE^−^ strain, and Russian lapinized vaccine strain [22,32,43,44,45,46,47,48].

C-strain, known as the Chinese hog cholera lapinized virus (HCLV), is an excellent attenuated vaccine that effectively protects pigs against CSF [49]. It was developed by Chinese scientists as hundreds of passages of a highly virulent CSFV in rabbits [22]. The C-strain can be used for emergency vaccination since it can induce rapid-onset and complete protection against CSF as early as 3 to 5 days after vaccination [50]. In China, the production of C-strain is constantly updated. C-strain was initially produced in rabbits, followed by primary calf testicular cells. Currently, this commercial vaccine is widely produced in the continuous swine testicular (ST) or porcine kidney (PK-15) cell line, which reduces the production cost of expensive primary cells and improves antigen yields and purity. CSF has been effectively prevented and controlled in many countries for nearly 70 years by massive vaccination with C-strain. In addition, the live attenuated vaccine strain GPE^−^ was produced by multiple passages of the virulent ALD strain in cells of swine, bovine, and guinea pig origins. However, the prolonged viremia and typical signs of CSF were observed after the GPE^−^ vaccine virus was readapted to pigs by serial passaging. Compared with the virus before passages, amino acid substitutions in E2 (T830A) and NS4B (V2475A and A2563V) are responsible for the pathogenicity of the GPE^−^/P-11 virus generated by reverse genetics [51].

C-strain has several disadvantages. It does not enable DIVA, which is necessary for CSF eradication when vaccination is implemented; immunization failure of C-strain usually occurs in case of maternally derived antibodies and persistent CSFV infection; and C-strain shows antigenic differences from emerging subgenotypes of CSFV [52]. In addition, C-strain replicates inefficiently in cell cultures, resulting in low virus titers, with an additional decline during vaccine production, storage, and freeze-drying.

### 2.2. E2-Based CSF Subunit Vaccines

Since the major neutralizing epitopes of CSFV are located on the E2 and E^rns^ glycoproteins, which induce NAbs against CSFV, the safe CSF subunit vaccines based on the E2 or E^rns^ glycoprotein were developed [53,54,55,56,57]. So far, the recombinant baculovirus inactivated vaccine of the CSFV E2 glycoprotein (Rb-03 strain) developed by Tecon Biologicals Co. Ltd. has been licensed (http://www.moa.gov.cn/govpublic/SYJ/201801/t20180115_6134895.htm, accessed on 25 December 2017). The vaccine is the first DIVA subunit vaccine of CSF in China that has good safety. Recently, the recombinant subunit vaccine based on the CSFV E2 glycoprotein expressed in HEK293T cells was comprehensively evaluated and commercialized (293T-E2, http://www.xmsyj.moa.gov.cn/zwfw/202209/t20220928_6412053.htm, accessed on 27 September 2022). However, subunit vaccines cannot completely prevent horizontal and vertical transmission, and the phenomenon still exists after the first or even the second immunization. Moreover, the production cost of subunit vaccines based on the CSFV E2 glycoprotein is higher than that of conventional LAVs. Therefore, it is not conducive to large-scale production. In addition, a major drawback of subunit vaccines is the relatively long period required to induce a protective response, which limits their use for emergency vaccination. Currently, only LAVs are able to confer complete protection for animals as early as 5 days post-immunization. Therefore, the shortcoming of the induction of rapid-onset and complete protection should be met for a CSF subunit vaccine to be applied for disease control.

The experimental E2-CD154 subunit vaccine based on the CSFV E2 fused with CD154 can confer long-lasting protection and provide complete protection against virulent CSFV challenge at 7 days post-immunization, even in the absence of NAbs, suggesting that CD154 could be considered as one of the best tools for the development of marker subunit vaccines [58]. The modified E2 containing a novel E2 signal peptide was secreted efficiently and presented with significantly improved immunogenicity compared with conventional E2-based vaccines. Moreover, a single dose of as low as 5 μg of the modified E2 protein protected piglets against lethal challenge. Its DIVA potential and protection suggest that the novel subunit vaccine based on CSFV E2-CD154 is a promising alternative to the live attenuated vaccine for developing countries [59,60,61].

A recombinant adenovirus vaccine rAdV-SFV-E2 expressing the CSFV E2 glycoprotein using replication-deficient adenovirus type 5 as a vector was developed. The results from animal experiments showed that the immunized pigs were completely protected against the highly virulent CSFV strain Shimen, and a high level of NAbs was detected in the immunized pigs. Subsequently, Xia and colleagues demonstrated that the adjuvant based on *Salmonella enteritidis*-derived bacterial ghosts (BG) could enhance the protective immunity of rAdV-SFV-E2 in pigs [62,63].

To address the issues of scalability, cost of production, and immunogenicity, Laughlin and colleagues reported an oil-in-water emulsion-adjuvanted, plant-made CSFV E2 subunit vaccine. The authors employed an Agrobacterium-mediated transient expression platform in *Nicotiana benthamiana* and formulated the purified antigen in novel oil-in-water emulsion adjuvants. The vaccine provided complete protection in challenged pigs, even after single-dose vaccination, which was accompanied by robust NAb responses [64]. Immunization of domestic pigs with a DNA vaccine encoding the full-length E2 protein of CSFV conferred complete protection against a lethal CSFV challenge [65].

### 2.3. Engineered Marker CSF Vaccines Using Reverse Genetics

While prophylactic vaccination is usually prohibited in disease-free countries with industrialized pig production, emergency vaccination is still considered. Marker vaccines are preferred, as they can allow DIVA and reduce the trade impacts [22]. Notably, the livestock vaccinated with the marker vaccines can avoid the economic loss caused by the improper culling during the implementation of the “stamping-out policy” for CSF.

Since the gene exchange between heterologous pestiviruses does not affect the viability of the chimeras, and there are essential differences between chimeric viruses and wild-type (wt) viruses in the coding proteins. Chimeric marker vaccines are attractive [66,67,68,69,70,71]; the chimeric vaccine “CP7_ E2alf” (Suvaxyn^®^ CSF Marker, Zoetis) harboring the *E2* gene of the CSFV Alfort/187 strain protected against transplacental transmission of moderately virulent CSFV [71]. The chimeric BVDV carrying the *E2* gene of the CSFV Alfort/187 strain could protect pigs from lethal CSFV challenge [68]. Remarkably, this marker vaccine can protect against transplacental transmission of moderately virulent CSFV [71]. In addition, the *E^rns^* gene of CSFV could be exchanged with that of other distant pestiviruses, such as Norway rat and pronghorn pestiviruses (Table 2) [66].

The LAVs can be further engineered into marker vaccines (Table 2) [71,77,78,79,80]. It is well-known that C-strain plays an important role in the prevention and control of CSF. The lack of a DIVA marker in C-strain poses a huge challenge for the eradication of CSF. At this stage, many scientists have made great efforts in this regard and carried out a series of research work aimed at engineering C-strain into a marker vaccine strain to achieve the purpose of DIVA. For example, the double marker live attenuated CSFV strain FlagT4v was developed [78]. Remarkably, experimental non-transmissible vaccines by trans-complementation of the E^rns^ or E2 of CSFV are potential marker vaccines for CSF [73]. In addition, the epitopes recognized by monoclonal antibodies (MAbs) have been mutated to generate marker vaccine candidates (33, 69, 75, 78). Our group also generated three C-strain-based marker CSFVs, rHCLV-E2F117A, rHCLV-E2G119A, and rHCLV-E2P122A, by mutation of the HQ06-recognized conserved linear epitope, which is able to induce weak NAbs [75]. The inactivated BVD vaccine can serve as a “marker” vaccine since anti-NS3 antibodies are low or undetectable following vaccination [81].

Successful implementation is dependent on a reliable accompanying diagnostic assay that allows DIVA. As induction of a protective immune response relies on virus-NAbs against the E2 protein of CSFV, the most promising DIVA strategy is based on the detection of E^rns^-specific antibodies in infected pigs [82].

### 2.4. Other Experimental Vaccines against CSF

The construction of genetically engineered subunit vaccines based on cell expression platforms has become a research hotspot in domestic biological product companies. The human replication-deficient adenovirus type 5-vectored CSF vaccine can induce complete immune protection, and the immune efficacy is comparable to that of conventional attenuated vaccines. It combines the safety of inactivated vaccines with the efficacy of LAVs [83]. Other new genetically engineered CSF vaccines under development mainly include DNA vaccines, gene-deleted vaccines, and synthetic peptide vaccines. Synthetic peptide vaccines can induce the production of CSFV-specific NAbs, but they cannot provide full protection due to the variations of antigenic epitopes between different CSFV genotypes [84].

Ding and colleagues constructed a combined vaccine based on the E2 protein of CSFV and the spike protein S1 subunit of porcine epidemic diarrhea virus (PEDV). The combined vaccine showed that good compatibility exists between the E2 and S1 antigens, and the E2-S1 vaccine can elicit a robust Th2-type cell-mediated humoral immune response [85].

### 2.5. LAVs and Inactivated Vaccines against BVD

At present, inactivated BVD vaccines and conventional attenuated BVDV strains, including NADL strain, Singer strain, Oregon C24V strain, and NY-1 strain, are commercially available (Table 1). In general, both are effective against homologous strains or strains with less antigenic differences. However, these vaccines cannot confer effective protection from heterologous BVDV strains [41,42]. The LAVs are safe for pregnant cows. However, there is a potential risk of transplacental infection of the fetus, which can easily cause fetal damage or persistent infection, immunosuppression, and mucosal disease. BVD LAVs could also be contaminated with non-cytopathic BVDV during the preparation process, which may cause disease in cattle after immunization [86]. In addition, other biological products, such as fetal bovine sera, may also be contaminated with BVDV and facilitate BVDV transmission to cattle. Immunization of pregnant animals with an attenuated BVD vaccine can cause fetal abortion or impede fetal immune system development [87]. Generally, infected or pregnant cows are not recommended for immunization with attenuated BVD vaccines. Considering these risks, the LAVs cannot be used widely.

Although the inactivated vaccine has great advantages in terms of safety, it does not induce complete protection (including homologous and cross-protection). For instance, Makosey and colleagues conducted immune tests on cattle with the BVDV-1 inactivated vaccine. They conducted challenge tests with BVDV-2, showing that inactivated vaccines of different genotypes of BVDV could only produce a certain degree of cross-protection with each other [41]. Because of the antigenic diversity of both BVDV-1 and BVDV-2, live attenuated and inactivated BVD vaccines containing strains of both genotypes have been developed for controlling BVDV infections [39,88,89,90]. The attenuated strains of BVDV can induce a broader and more durable immune response than inactivated vaccines, possibly due to the involvement of a more potent T-cell-mediated immune response [91,92]. The inactivated BVD vaccines are safe and formulated in an adjuvant to induce adequate immunity [93,94,95]. The duration of immunity induced by inactivated BVD vaccines tends to be shorter, and the antibody response against different strains or isolates may not be adequate for protection compared to live vaccines [91,96].

The etiological agents of the bovine respiratory disease complex include BVDV, infectious bovine rhinotracheitis virus (IBRV), parainfluenza virus type 3 (PIV3), bovine respiratory syncytial virus (BRSV), and bacterial pathogens [97]. BVD vaccine strains are also formulated and administrated with these respiratory pathogens. Therefore, developing a marker or multivalent vaccine is an effective way to prevent viral infections.

### 2.6. Other Experimental Vaccines against BVD

Various BVD vaccines have been developed using reverse genetics [98]. BVDV with 5′ UTR deletion is able to produce high-level anti-BVDV NAbs, protecting cattle from homologous challenge [99]. However, there may be recombination between the chimeric viruses and wt pestiviruses, resulting in unpredictable properties. The efficacy and safety of this chimeric pestivirus vaccine against BVDV genotypes 1 and 2 have been confirmed in numerous vaccination challenge trials [100].

The candidate vaccine was further attenuated by deletion of the N^pro^ protein, a type I interferon antagonist. Immunization of cattle with the chimeric vaccine virus BuPV_Δ*N^pr^*^o^_*E1E2* CP7,the major immunogenic E1 and E2 of BuPV were substituted by the heterologous E1 and E2 of the BVDV-1 strain CP7, (modified live or inactivated) followed by subsequent experimental challenge infections confirmed the safety of the prototype strain. It provided a high level of clinical protection against BVDV-1 [101].

Another approach is the targeting of E2 to major histocompatibility type II molecule (MHC-II)-expressing cells. The strategy of the vaccine APCH-E2 is the fusion of the BVDV E2 protein to a single-chain antibody, APCH. The APCH targets the E2 antigen to the MHC-II present on antigen-presenting cells, and this new subunit vaccine could induce rapid and sustained NAbs compared with a conventional vaccine in cattle [102].

Although the above experimental (or prototype) vaccines have demonstrated good protective efficacy, to become commercial vaccines, the following six criteria need to be met: (1) DIVA; (2) The attenuated vaccines do not spread or revert to virulence; (3) They are genetically stable to target animals and non-target animals; (4) The production process of commercial vaccines should be simple under normal conditions; (5) They should induce immune protection quickly and life-long immunity; (6) They should protect from challenge with different CSFV strains.

### 2.7. Cross-Protection against Different Subgenotypes Conferred by Pestivirus Vaccines

CSFV has only one serotype but can be divided into three groups with three or four subgenotypes: 1.1 to 1.3, 2.1 to 2.3, and 3.1 to 3.4 [103]. C-strain belongs to subgenotype 1.1, but it can induce cross-protection against different highly virulent strains and various genotypes [103,104]. In addition, more and more studies have shown that C-strain can provide cross-protection against different subgroups and partial or complete cross-protection between different viruses [38,55,105,106].

BVDV can be divided into three groups: BVDV-1 (1a-1t), BVDV-2 (2a-2c), and BVDV-3 [107]. Similarly, some LAVs of BVDV can also provide cross-protection for different species or subtypes of pestiviruses [42,108]. The vaccine based on the BVDV-1a and BVDV-2 strains provided 96% protection from persistent fetal infection caused by the BVDV-1b strain [109]. A commercially available BVD LAV can provide complete protection against BVDV-1 and BVDV-2 up to one year after a single vaccination. The BVD vaccine might elicit partial protection against the HoBi-like pestivirus. However, due to the genetic and antigenic variability among different BVDV strains, a vaccine effective in one region may fail to protect against infections caused by different virus strains in another region [110]. No BVD vaccines developed with the predominant strains in China are available. An inactivated BVDV 1a NM01 vaccine strain was evaluated by challenge with the Chinese BVDV 1b JL strain. The clinical symptoms, such as the temperature and leukopenia of the immunized calves and viral shedding, were significantly less than the mock immunized calves after challenging with the virulent BVDV 1b strain, indicating that the BVDV 1a vaccine strain elicited efficacious protection against the endemic BVDV 1b strain in China [111]. Hamers and colleagues showed that an inactivated BVDV genotype I vaccine conferred protection against genotype II (890 strain) BVDV challenge, resulting in a significant reduction in clinical symptoms, serological response, and viremia [112]. It is calculated that multiple conservative sequences exist in the pestiviral genome, including antigen epitopes. The attenuated vaccine strains contain the E2 and E^rns^ epitopes of prevalent strains, ensuring the vaccine efficacy. Whether to further develop a universal pestivirus vaccine through these sequences is also being investigated. The immune system has a more comprehensive recognition of the virus than subunit vaccines, including more conservative structural proteins. Antibodies induced by these structural proteins can also play a role in cross-protection [113]. The cross-protection level is relatively low for inactivated vaccines or LAVs. Therefore, researchers still need to select the strains with good immunogenicity and a broad antigenic spectrum as a vaccine to improve the vaccine efficacy.

## 3. Development of Multivalent Vaccines Based on Pestiviruses

**Figure 1:**
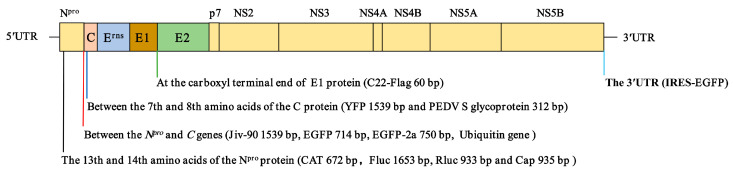
Schematic diagram of the foreign gene insertion in the pestiviral genome. The insertion sites and sizes of foreign genes in the pestiviral genome are shown. The foreign genes can be inserted between the 13th and 14th amino acids of the N^pro^ protein, downstream of the N^pro^ protein and the 7th and 8th amino acids of the C protein, between E1 and E2, and upstream of the 3′ UTR of the pestiviral genome.

At present, various foreign genes of varied sizes have been engineered into different positions of the pestiviral genome (Figure 1), suggesting that pestiviruses can be used as vectors to deliver foreign genes. The pestiviral genome replicates in the cytoplasm, and the viral RNA cannot be integrated into the host cell genome, ensuring the biosafety of pestiviruses. However, whether a pestivirus can be used as a classic viral vector to express foreign genes needs further exploration. The marker pestivirus vaccines such as engineered marker C-strain or CP7_E2alf can be used as viral vectors for developing bivalent/multivalent vaccines. Further studies are needed to determine whether the LAV-based recombinant vaccines expressing foreign genes are safe.

### 3.1. The Sites Suitable for the Insertion of Foreign Genes

Various strategies associated with foreign gene expression have been attempted. A widely used expression strategy is to fuse the foreign gene with one of the viral proteins. It was reported that the gene coding for bacterial chloramphenicol acetyltransferase (CAT) could be inserted into the viral *N^pro^* gene of the CSFV strain Alfort/187 [114]. Then, EGFP, Fluc, and Rluc were fused to the viral N^pro^ protein of the CSFV Shimen strain, respectively [75,76,77]. It has been shown that the *N^pro^* and *C* genes of CSFV can allow the insertion of foreign genes [114]. Our previous study also demonstrated that the EGFP gene could be inserted between the 13th and 14th amino acids of the N^pro^ protein of the highly virulent CSFV Shimen strain [115]. Based on the safety and efficacy of attenuated vaccines against pestiviral infections, these vaccine strains have great potential as viral vectors for developing multivalent vaccines to control co-infections in pigs. 

The vaccines based on viral vectors can activate the humoral and cellular immunity of the hosts [62,116,117,118]. For example, the double-strand RNA produced during genome replication can enhance the efficacy [119,120]. The foreign genes in the viral vector are expressed continually and induce persistent immunity [121]. The functions of foreign proteins are minimally affected due to the smaller size of the structural proteins.

However, this is no evidence that this strategy can be suitable for C-strain. The foreign gene can be expressed separately by introducing an internal ribosome entry site (IRES) or the foot-and-mouth disease virus (FMDV) 2A self-cleaving peptide, which can undergo self-cleavage and not affect the expression of other proteins on the vector. For example, a recombinant C-strain expressing the PCV2 Cap by 2A-mediated cleavage was generated and evaluated in vitro and in vivo [122]. Notably, the same strategy might lead to different effects on the growth of the same virus due to the different insertion sites. For example, the use of the 2A peptide to achieve expression of a separate reporter might constitute a promising approach as the 2A peptide is small and can readily be self-cleaving while minimizing the possibility of the loss of functions of the viral proteins.

It is well-documented that the *N^pro^* gene is not required for CSFV replication, and the CSFV mutant lacking the full-length *N^pro^* gene is attenuated in pigs [123,124,125]. Remarkably, the CSFV mutant with the *C* gene deleted and the *NS3* gene mutated was rescued and attenuated, suggesting that the *C* gene was dispensable for viral replication and able to be replaced by foreign genes [126]. Furthermore, the *E^rns^*-deleted CSFV mutant was developed as a potential non-transmissible, live-attenuated marker vaccine [72]. Therefore, foreign genes might be introduced by substituting the CSFV *N^pro^*, *C*, or *E^rns^* genes.

The EGFP and FMDV 2A fusion gene was inserted between the *N^pro^* and *C* genes of the noncytopathic BVDV strain SD1. In addition, the reporter virus was similar to wt SD1 in viral RNA replication and protein expression and comparable to wt SD1 in growth kinetics; however, this virus had a peak virus titer approximately 0.5 log_10_ lower and a maximum yield about 4 h later than wt SD1. The study has indicated that BVDV is a suitable viral vector for the stable expression of heterologous genes when inserted between the *N^pro^* and *C* genes [127]. The foreign gene encoding the PEDV S glycoprotein was inserted between the seventh and eighth amino acids of the C protein of the BVDV genome by homologous recombination vASH-dS312, which could successfully express the PEDV S glycoprotein. The immunized mice were healthy and showed no clinical symptoms. The antigen S499-602 was inserted into the infectious cDNA clone pASH28 of pig-originated BVDV-2 in tandem by overlapping PCR, located between the seventh and eighth amino acids of the C protein. IgG antibodies against BVDV and PEDV could be detected in the mice administered with vASH-dS312 by intramuscular injection, which had neutralizing activity against BVDV and PEDV [128]. However, none of these experimental vaccines have been subjected to an immune evaluation in pigs, and thus it remains unknown whether they can provide protection.

### 3.2. The Size and Genetic Stability of the Inserted Foreign Genes

Though much smaller than the genomes of popular vectors like PRV and vaccinia virus, the pestiviral genome also allows the insertion of foreign genes. EGFP (714 bp), Fluc (1653 bp), and Rluc (933 bp) have each been inserted into the genome of the CSFV Shimen strain [115,129,130]. The insertion of 1,539 bp was achieved in the CSFV strain Alfort-p447, and the insertion derived from the cytopathic BVDV strain CP8 encoded a 513-aa fusion peptide, encompassing fragments from viral sequences (C, E^rns^, and N^pro^) and cellular sequences [JivI, JivII, and bovine homolog to human nuclear protein Hcc-1 (Hcc-1 *)] [131]. Whether the attenuated vaccine strains (such as C-strain) can tolerate similar insertions warrants experimentation. Since the pestiviral genome is relatively small, the delivery of antigens with smaller molecular weights may be more suitable for pestiviruses.

### 3.3. The Expression Levels of Foreign Genes in the Pestiviruses

The expression levels of the foreign genes in the pestiviral vectors can be affected by various factors, such as the insertion sites and expression strategy. For example, the titers of the engineered C-strain and its mutants are relatively low, which might affect the expression levels of foreign genes. The robust adaptation of the attenuated viruses to the cells and the manufacturing techniques are required to improve the viral titers [132].

Our group has generated three C-strain-based recombinant viruses expressing the capsid (Cap) gene of PCV2 by reverse genetic manipulation (Figure 1). The Cap protein is a major immunogenic protein of PCV2, which can induce protective immunity [133]. The data showed that rHCLV-uspCap and rHCLV-pspCap rather than rHCLV-2ACap elicited detectable anti-Cap antibodies in rabbits, which demonstrated that C-strain could be used as a viral vector to develop bivalent vaccines [122]. Furthermore, it has been shown that the recombinant BVDV expressing the PEDV spike protein, as a recombinant virus vector, can induce higher titers of NAbs and provide protection against virulent challenge [128,134].

## 4. The Limitations and Prospects of Pestivirus Vaccines

In summary, a number of vaccine strategies have been explored to combat pestivirus infections of livestock and wildlife. However, there are several limitations in the pestivirus vaccines. Firstly, the safety of engineered vaccines based on pestiviral vectors should be evaluated in the field. This is relevant for vaccination against infectious diseases and potentially exploiting virus-based vectors in vaccination strategies where individuals are sometimes immunocompromised. Secondly, maternally derived antibodies may inhibit the immune responses of LAVs. Thirdly, subunit vaccines usually cannot provide rapid-onset and complete protection. Additionally, new-type pestivirus vaccines need a long time before they can be commercially available.

Replicons are replication-competent RNA molecules that are incapable of generating infectious progeny viruses due to the loss of one or more structural proteins. The genome sequences encoding NS3-NS5B together with the 5′ and 3′ UTRs are the minimal elements required for autonomous pestiviral RNA replication [135]. Significantly, the viral E2-coding region can enhance the replication efficiency of the CSFV RNA replicon [136]. The RNA replicon activates the cellular immunity of the hosts and has potential as a vector to express foreign genes. For instance, chimeric CSF-Japanese encephalitis (JE) viral replicon as a non-transmissible vaccine candidate has been proven effective against CSFV and JEV infections [137]. Other studies have confirmed that activated specific cellular immunity (CD8^+^/CD4^+^ T cells) contributes to the protection against ASFV infection [138,139]. Considering the safety and efficacy of the pestivirus RNA replicons, multivalent vaccines can be developed based on the RNA replicons.

Viral vectors hold great promise for development of multivalent vaccines to counter co-infectious diseases. Compared with other viral vectors, pestivirus-based vectors have the following advantages: (1) The infectious clones of the RNA viruses are easier to be engineered than those of the DNA viruses due to their smaller genome size of 7 to 19 kb; (2) The double-stranded RNA produced by the genome replication can enhance the immunization efficacy; (3) Viral proteins are expressed with high efficacy; (4) The replication of the RNA occurs in the cytoplasm rather than in the nucleus, thus avoiding the RNA degradation; (5) No RNA can be integrated into the DNA of the host; (6) Since they have fewer viral structural proteins and cause less immune responses against the vector, the pestiviral vectors have great potential for developing safe and effective vaccines against the animal diseases.

Several CSF vaccines have been developed for oral immunization of wild boars [140,141,142,143,144,145,146,147,148]. For example, immunization with an oral bait vaccine based on C-strain proved to be safe and efficacious. It has been demonstrated that CP7_E2alf is a safe and efficient marker vaccine strain for oral immunization of wild boars against CSF [106,149,150]. New immunization regimes of pestivirus vaccines may be developed in the future.

Collectively, various pestivirus vaccines have been developed by different attractive strategies and have shown the advantages and disadvantages in terms of the safety, efficacy involved in the rapid-onset protection, cross-protection, and DIVA potential, which will provide new insights into the development of novel vaccines against emerging pestiviruses.

## Figures and Tables

**Table 1:** Commercially available vaccines against pestiviral infections.

Vaccines	Development Strategies	Advantages	Disadvantages	References
C-strain	Hundreds of passages of a highly virulent classical swine fever virus (CSFV) in rabbits	Safety, rapid-onset protection (3 to 5 days post-vaccination), long-lasting protection (six months)	No DIVA, maternal antibody interference	[35]
CSFV E2	Baculovirus-expressed E2	Safety, DIVA	No complete prevention from vertical transmission, a relatively long period of time required to induce a protective response, and the need for a booster immunization to achieve a full protection.	[36]
CP7-E2alf	BVDV harboring the *E2* gene of the CSFV Alfort/187 strain	DIVA, partial protection against early CSFV challenge	The neutralizing antibodies (NAbs) produced are lower than those of C-strain	[37,38]
BVDV inactivated vaccine	Upgraded by processes such as concentration and purification, and emulsified biphasic oil emulsion adjuvant	Higher protection, longer duration of immunity	No DIVA	[39,40,41]
BVDV LAVs	Isolation of naturally attenuated strains, more than 60 passages of a highly virulent strain in the MDBK cells, or gene-deletion attenuated strains	High-level NAbs	Safety risk, no DIVA	[42]

**Table 2:** LAV-based marker CSF vaccines.

Marker Strains	Development Strategies	References
Fl22	Deletion of 66 amino acids in *E^rns^*	[72]
Fl23	Deletion of 215 amino acids in *E^rns^*	
Flc9	Replacement of the N-terminal half of E2 of C-strain by that of BVDV	[67]
Flc11	Replacement of the *E^rns^* gene of C-strain by that of BVDV	
Flc4	Deletion of the B/C region of *E2* (aa 693 to 746)	[73]
Flc47	Deletion of the whole *E2* gene (aa 689~1062)	
Flc48	Deletion of the A region (aa 800 to 864) of *E2*	
Flc-LOM-BE^rns^	Replacement of the CSFV *E^rns^* gene and the 3′-end (52 bp) of the CSFV *C* gene with the corresponding BVDV genes	[74]
rHCLV-E2P122A	Mutation of the epitopes in E2	[75]
vGPE^−^	Ten amino acids of substitutions were recognized, compared with the original GPE^−^ vaccine	[76]
